# A Metric Based on the Efficient Determination Criterion

**DOI:** 10.3390/e26060526

**Published:** 2024-06-19

**Authors:** Jesús E. García, Verónica A. González-López, Johsac I. Gomez Sanchez

**Affiliations:** Department of Statistics, University of Campinas, Campinas 13083-859, São Paulo, Brazil; jg@ime.unicamp.br (J.E.G.); or j216401@dac.unicamp.br (J.I.G.S.)

**Keywords:** partition Markov models, Bayesian information criterion, entropy

## Abstract

This paper extends the concept of metrics based on the Bayesian information criterion (BIC), to achieve strongly consistent estimation of partition Markov models (PMMs). We introduce a set of metrics drawn from the family of model selection criteria known as efficient determination criteria (EDC). This generalization extends the range of options available in BIC for penalizing the number of model parameters. We formally specify the relationship that determines how EDC works when selecting a model based on a threshold associated with the metric. Furthermore, we improve the penalty options within EDC, identifying the penalty ln(ln(n)) as a viable choice that maintains the strongly consistent estimation of a PMM. To demonstrate the utility of these new metrics, we apply them to the modeling of three DNA sequences of dengue virus type 3, endemic in Brazil in 2023.

## 1. Introduction

This article embarks on an exploration of the efficient determination criterion (EDC), as introduced in [1], with a particular emphasis on formulating an EDC-based metric. Our endeavor is bolstered by the presence of a Bayesian information criterion (BIC) metric proposed in [2], designed to provide consistent estimations of partition Markov models [2]. Our aim is to extend the scope of the BIC-based metric, thereby broadening the array of algorithms available for identifying partition Markov models.

To achieve our goal, we furnish a theoretical framework delineating the operational principles underlying the BIC/EDC, the BIC-based metric, and additionally, we conduct a brief survey of the current research landscape within this domain to provide context for our approach.

Let $\left(X_{t}\right)$ be a discrete-time order *o* Markov chain on a finite and discrete alphabet Δ, with o<∞; let us call $\Omega =\Delta^{o}$ the state space. Denote the string $a_{k}a_{k+1}\ldots a_{m}$ by $a_{k}^{m},$ where $\;a_{i}\in \Delta ,\;k\leq i\leq m.$ For each a∈Δ and s∈Ω, the transition probability from the state *s* to *a* is

(1) $$\begin{array}{c} P\left(a|s\right)=\text{Prob}\left(X_{t}=a|X_{t-o}^{t-1}=s\right). \end{array}$$

Let $P=\{\Gamma_{1},\Gamma_{2},\ldots ,\Gamma_{\left|P\right|}\}$ be a partition of Ω, then for each pair of parts $\Gamma_{i}$ and $\Gamma_{j},\;\;i\neq j,$ i,j∈{1,…,|P|}, $\Gamma_{i}\cap \Gamma_{j}=\emptyset$, and $\Omega =\cup_{i=1}^{\left|P\right|}\Gamma_{i}.$

Note that a part Γ of the partition is constituted by a collection of states coming from Ω; we reformulate the notion introduced by Equation (1) as follows, for a∈Δ,Γ∈P:

(2) $$\begin{array}{ccc} P(\Gamma ,a) & = & \sum_{s\in \Gamma }\text{Prob}\left(X_{t-o}^{t-1}=s,X_{t}=a\right),\\ P(\Gamma ) & = & \sum_{s\in \Gamma }\text{Prob}\left(X_{t-o}^{t-1}=s\right),\\ P(a|\Gamma ) & = & \frac{P(\Gamma ,a)}{P(\Gamma )}\;\;\;\;\text{if}\;\;\;\;P\left(\Gamma \right)>0. \end{array}$$

Given the previous notation, we appeal to a model in $\left(X_{t}\right)$ which allows a more efficient estimation of the transition probabilities, introduced by Equation (1); see [2].

**Definition** **1.** *Let $\left(X_{t}\right)$ be a discrete-time order o Markov chain on a finite and discrete alphabet Δ,o<∞. Two states $s,r\in \Omega =\Delta^{o}$ are equivalent (denoted by $s{∼}_{p}r$) if P(a|s)=P(a|r)∀a∈Δ. For any s∈Ω, the equivalence class of s is given by the set of states $\{r\in \Omega :r{∼}_{p}s\}.$*

The previous notion allows the definition of a Markov chain with minimal partition P, that is, one which follows the equivalence relationship.

**Definition** **2.** *Let $\left(X_{t}\right)$ be a discrete-time order o Markov chain on a finite and discrete alphabet Δ,o<∞, and let $P=\{\Gamma_{1},\Gamma_{2},\ldots ,\Gamma_{\left|P\right|}\}$ be a partition of $\Omega =\Delta^{o}$; $\left(X_{t}\right)$ is a Markov chain with minimal partition P if P is defined by the relationship ${∼}_{p}$ introduced by Definition 1.*

As previously indicated, the objective of this model is to allow a more efficient estimation of the probabilities introduced by Equation (1), which occurs in the most efficient way possible by identifying the parts of the minimal partition (Definition 2), and thus, being able to use all the states inserted in each part to estimate a single probability per part. To identify the partition P introduced in Definition 2, a strategy must be implemented as shown below.

In a given sample $x_{1}^{n},$ of size n, coming from the stochastic process $\left(X_{t}\right)$ under the assumptions of Definition 2, given the state s∈Ω and the element of the alphabet a∈Δ, we denote the number of occurrences of *s* followed by *a* in the sample $x_{1}^{n}$ by $N_{n}\left(s,a\right)=\left|\left\{t:o<t\leq n,x_{t-o}^{t-1}=s,x_{t}=a\right\}\right|$ and $N_{n}\left(s\right)=\sum_{a\in \Delta }N_{n}\left(s,a\right)$ is the number of occurrences of *s* in the sample $x_{1}^{n}.$ Also, given a partition P of Ω, denote the number of occurrences of elements into Γ (part of P) followed by *a* as

(3) $$\begin{array}{c} N_{n}\left(\Gamma ,a\right)=\sum_{s\in \Gamma }N_{n}\left(s,a\right),\;\;a\in \Delta , \end{array}$$

the accumulated number of values $N_{n}\left(s\right)$ for s∈Γ is denoted by

(4) $$\begin{array}{c} N_{n}\left(\Gamma \right)=\sum_{s\in \Gamma }N_{n}\left(s\right). \end{array}$$

Note that $N_{n}\left(\Gamma ,a\right)$ and $N_{n}\left(\Gamma \right)$ can be computed for any partition P of Ω, not only for the partition introduced by Definition 2.

The counts of occurrences, in this case $N_{n}\left(\Gamma ,a\right)$ and $N_{n}\left(\Gamma \right)$, allow the estimation of probabilities (Equation (2)) subject to a modification of the likelihood function of the sample. The likelihood of the sample is

$$P\left(x_{1}^{n}\right)=P\left(x_{1}^{o}\right)\prod_{a\in \Delta ,\Gamma \in P}P{\left(a|\Gamma \right)}^{N_{n}\left(\Gamma ,a\right)},$$

then, the maximum of the modified log-likelihood is

$$\sum_{a\in \Delta ,\Gamma \in P}N_{n}\left(\Gamma ,a\right)\ln\left(\frac{N_{n}\left(\Gamma ,a\right)}{N_{n}\left(\Gamma \right)}\right),\;\;\;\text{with}\;\;\;N_{n}\left(\Gamma \right)>0,\forall \Gamma .$$

And,

(5) $$\begin{array}{c} \frac{N_{n}\left(\Gamma ,a\right)}{N_{n}\left(\Gamma \right)},\;\;\;\text{with}\;\;\;N_{n}\left(\Gamma \right)>0, \end{array}$$

is the maximum likelihood estimator of P(a|Γ) given in Equation (2).

As shown in [2], under the assumptions of Definition 2, the partition P can be consistently (strong consistency) retrieved using the Bayesian information criterion (BIC), defined as

(6) $$\begin{array}{c} \text{BIC}\left(x_{1}^{n},P\right)=\sum_{a\in \Delta ,\Gamma \in P}N_{n}\left(\Gamma ,a\right)\ln\left(\frac{N_{n}\left(\Gamma ,a\right)}{N_{n}\left(\Gamma \right)}\right)-\frac{\left(|\Delta |-1)|P\right|}{\alpha }\ln\left(n\right), \end{array}$$

with α>0, a constant value. Then, the BIC takes into consideration the maximum of the modified log-likelihood term penalized by $\frac{\left(|\Delta |-1)|P\right|}{\alpha }\ln\left(n\right)$, where (|Δ|−1)|P| is the number of probabilities to be estimated.

In practice, candidates to be the partition according to Definition 2 are compared, and the partition with the higher BIC value is considered more suitable. Also, in [2] a metric is introduced based on the BIC criterion, along with clustering algorithms, which are used to obtain P; the metric is defined below. To achieve consistent estimation, such a metric operates on partitions of the state space that follow certain rules. The metric is then able to refine partitions until it identifies the one cited by Definition 2. The partitions in which we will apply the metric, are made up of members (parts) formed by states sharing all the transition probabilities. The definition to follow formalizes the concept.

**Definition** **3.** *Let $\left(X_{t}\right)$ be a Markov chain of order o, with finite and discrete alphabet Δ,o<∞, and state space $\Omega =\Delta^{o}.$ Set a partition of $\Omega ,\;\;P=\{\Gamma_{1},...,\Gamma_{\left|P\right|}\},$*

*i*. *given a part* Γ *of P,* Γ *is a good part if ∀a,a∈Δ, P(a|s)=P(a|r),∀r,s∈Γ,r≠s.*
*ii*. *P is a good partition of* Ω *if* Γ *satisfies i. ∀Γ∈P.*

Under the validity of Definition 3-i, the probabilities introduced by Equation (2) are

(7) $$\begin{array}{c} P\left(a|\Gamma \right)=\text{Prob}\left(X_{t}=a|X_{t-o}^{t-1}=s\right)\;\;\;\forall a\in \Delta ,\forall s\in \Gamma , \end{array}$$

since all the elements of the good part Γ of P share the transition probabilities. Note that the partition identified by Definition 2 verifies Definition 3-ii, but the reciprocal is naturally not valid. A straightforward example of a good partition is one composed of all the states being isolated.

The following introduces a notion used to estimate the minimal partition (Definition 2). This criterion operates on good parts (Definition 3-i).

**Definition** **4.** *Let $\left(X_{t}\right)$ be a Markov chain of order o, with finite and discrete alphabet Δ,o<∞, and state space $\Omega =\Delta^{o}$; $x_{1}^{n}$ is a sample of the process and let $P=\{\Gamma_{1},...,\Gamma_{\left|P\right|}\}$ be a good partition of Ω,*

$$\begin{array}{c} d_{P}\left(i,j\right)=\frac{\alpha }{\left(|\Delta |-1)\ln(n\right)}\sum_{a\in \Delta }\left\{\sum_{k\in \{i,j\}}N_{n}\left(\Gamma_{k},a\right)\ln\left(\frac{N_{n}\left(\Gamma_{k},a\right)}{N_{n}\left(\Gamma_{k}\right)}\right)-N_{n}\left(\Gamma_{ij},a\right)\ln\left(\frac{N_{n}\left(\Gamma_{ij},a\right)}{N_{n}\left(\Gamma_{ij}\right)}\right)\right\}. \end{array}$$

*where α is a constant and positive value, $N_{n}\left(\Gamma_{ij}\right)=N_{n}\left(\Gamma_{i}\right)+N_{n}\left(\Gamma_{j}\right),$ $N_{n}\left(\Gamma_{ij},a\right)=N_{n}\left(\Gamma_{i},a\right)+N_{n}\left(\Gamma_{j},a\right),\;\forall a\in \Delta .$*

In [2], it is proved that $d_{P}$ of Definition 4 is a metric, meaning that, if $\Gamma_{l}\in P,l\in \left\{i,j,k\right\},$

i. $d_{P}\left(i,j\right)\geq 0,$ with equality, if and only if $\frac{N_{n}\left(\Gamma_{i},a\right)}{N_{n}\left(\Gamma_{i}\right)}=\frac{N_{n}\left(\Gamma_{j},a\right)}{N_{n}\left(\Gamma_{j}\right)}\;\;\forall a\in \Delta ;$
ii.
$d_{P}\left(i,j\right)=d_{P}\left(j,i\right);$

iii.
$d_{P}\left(i,k\right)\leq d_{P}\left(i,j\right)+d_{P}\left(j,k\right).$

As a consequence of the property i of a metric, the ability of $d_{P}$ to operate adequately depends on the accuracy of the maximum likelihood estimation of the transition probabilities $P(a|\Gamma_{i})$ and $P(a|\Gamma_{j}),\;\;\forall a\in \Delta .$ That is, when the estimators of those probabilities $\frac{N_{n}\left(\Gamma_{i},a\right)}{N_{n}\left(\Gamma_{i}\right)},$ $\frac{N_{n}\left(\Gamma_{j},a\right)}{N_{n}\left(\Gamma_{j}\right)}\;\;\forall a\in \Delta$ are near and the sample size *n* is large enough, we have evidence of proximity between $P(a|\Gamma_{i})$ and $P(a|\Gamma_{j}),\forall a\in \Delta .$ And, such a finding indicates that the elements of both parts must be together.

Partition Markov models, commonly referred to as those delineated by Definition 2, have found application in diverse realms. For instance, they have been employed in data compression in conjunction with Huffman coding, as exemplified in [3]. Across these investigations, the utilization of the BIC-based metric $d_{P}$ has proven indispensable. Also, in [2], this metric has been pivotal for modeling the behavior of internet users. The partition Markov model allows identifying the chances of a user visiting a certain internet site in their next step, based on their history, and identifies equivalent histories in the sense introduced by Definition 2.

Since the support of $d_{P}$ is the BIC criterion, the question arises whether there is a broader criterion than BIC that is capable of maintaining strong consistency in the estimation of P. The next section shows that such a criterion exists (a generalization of BIC) and was proved by [4]. Then, the next question that we propose to answer is whether such a generalization of the BIC allows the creation of a metric that generalizes the one introduced in [2].

The next section (Section 2) addresses the problem by introducing the efficient determination criterion, and then, presenting how this criterion is linked to a metric, also introducing a cut-off point that enables the practical use of the metric based on the efficient determination criterion, for sufficiently large values of n. Section 3 shows an application in which different fits of model—Definition 2—are compared, inferred by variants of the efficient determination criterion, indicated as recommended in Section 2. This article ends with the Conclusions—Section 4—in which we highlight the main contributions, and the Bibliography section.

## 2. Efficient Determination Criterion

Ref. [1] proposes a criterion generalizing the BIC criterion, the efficient determination criterion (EDC). In that paper, the proposal is to introduce a sequence ${\left\{w_{n}\right\}}_{n\geq 1}$ in the place of ${\left\{\ln\left(n\right)\right\}}_{n\geq 1}$; see Equation (6). The generalization also offers more options in the penalty term of Equation (6), instead of the number of parameters a function γ(·) is introduced acting over the number of parameters; this function is strictly increasing in the number of parameters. Under the assumptions of Definition 2, the criterion is formulated as follows:

(8) $$\begin{array}{c} \text{EDC}\left(x_{1}^{n},P\right)=\sum_{a\in \Delta ,\Gamma \in P}N_{n}\left(\Gamma ,a\right)\ln\left(\frac{N_{n}\left(\Gamma ,a\right)}{N_{n}\left(\Gamma \right)}\right)-\gamma \left(\frac{\left(|\Delta |-1)|P\right|}{\alpha }\right)w_{n}. \end{array}$$

With α>0 a constant value, γ(·) being a strictly increasing function, and $\left\{w_{n}\right\}$ a sequence of positive numbers depending on n. As well as BIC, candidates to be the partition according to Definition 2 are compared, and the higher the EDC, the more indicated the partition is. Note that if we choose γ(·) as being the identity function, $\gamma \left(\frac{\left(|\Delta |-1)|P\right|}{\alpha }\right)=\frac{\left(|\Delta |-1)|P\right|}{\alpha }$, and $w_{n}=\ln\left(n\right),$ then Equation (6) is recovered. Then, clearly the EDC criterion is a generalization of the BIC criterion.

Ref. [4] proves that the EDC criterion provides a strongly consistent way to estimate the partition P of Definition 2 if

(9) $$\begin{array}{c} \lim_{n\to \infty }\frac{w_{n}}{n}=0\;\;\;\;\;\text{and}\;\;\;\;\lim_{n\to \infty }\frac{w_{n}}{\ln(\ln(n))}=\infty . \end{array}$$

**Figure 1:**
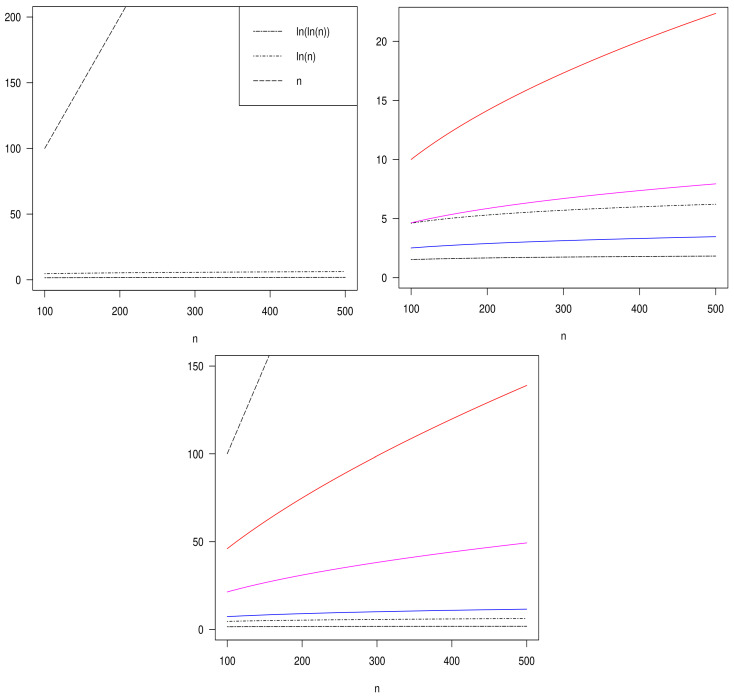
**Top left**: Double-dashed line, ln(ln(n)); dotted-dashed line, ln(n); long-dashed line, n. **Top right**: $w_{n}=n^{1/2}$ (in red); $w_{n}=n^{1/3}$ (in magenta); $w_{n}=n^{1/5}$ (in blue); double-dashed line, ln(ln(n)); dotted-dashed line, ln(n). **Bottom**: $w_{n}=n^{1/2}\ln\left(n\right)$ (in red); $w_{n}=n^{1/3}\ln\left(n\right)$ (in magenta); $w_{n}=n^{1/10}\ln\left(n\right)$ (in blue); double-dashed line, ln(ln(n)); dotted-dashed line ln(n); long-dashed line *n*.

Note that if we take $w_{n}=n^{a}$ for a∈(0,1), the conditions given in Equation (9) are valid. Also, we can use $w_{n}=a\ln\left(n\right)$ for a>0. Another option is to use $w_{n}=n^{a}\ln\left(n\right)$ for a∈(0,1). Figure 1 shows penalty functions $w_{n}$ verifying Equation (9). We see in the figure that functions $w_{n}$ are positioned between the functions *n* and ln(ln(n)). And between *n* and ln(ln(n)) is also the $w_{n}$ related to the BIC criterion ($w_{n}=\ln\left(n\right)$).

Clearly, the penalty ln(ln(n)) does not verify the second statement of Equation (9), but according to [5] it is an optimal penalty term for estimating the order of a Markov chain. With such inspiration in mind, the following proposition guarantees that ln(ln(n)) can also be used to obtain a consistent estimate of P. To state the proposition we introduce the notion of relative entropy.

**Definition** **5.** *Let P and Q be probability distributions on Δ. The relative entropy between P and Q is given by $D\left(P(\cdot )||Q(\cdot )\right)=\sum_{a\in \Delta }P\left(a\right)\ln\left(\frac{P(a)}{Q(a)}\right),$ with Q(a)≠0,∀a∈Δ.*

**Proposition** **1.** *Let $\left(X_{t}\right)$ be a Markov chain of order o, with finite and discrete alphabet Δ,o<∞, and state space $\Omega =\Delta^{o}$; $x_{1}^{n}$ is a sample of the process and let $P=\{\Gamma_{1},...,\Gamma_{\left|P\right|}\}$ be a partition of Ω, and P(·|Γ) be the probability given by Equation (2) related to a good part* Γ *(Definition 3-i). To any δ>0 there exists κ>0 (depending on P(·|·)) such that, eventually, almost surely as n→∞*

$$|\frac{N_{n}\left(\Gamma ,a\right)}{N_{n}\left(\Gamma \right)}-P\left(a|\Gamma \right)|<\sqrt{\frac{\delta \ln(\ln(n))}{N_{n}\left(\Gamma \right)}},$$

*for all Γ, good part, with $N_{n}\left(\Gamma \right)\geq 1$ and o<κln(ln(n)).*

**Proof.**  From the proof of Corollary 2 of [6] (on page 1621), we obtain that for any ϵ>0 there is κ>0 (depending on P(·|·)) such that, eventually, almost surely as n→∞

(10) $$|\frac{N_{n}\left(s,a\right)}{N_{n}\left(s\right)}-P\left(a|s\right)|<\sqrt{\frac{\epsilon \ln(\ln(n))}{N_{n}\left(s\right)}},$$

for all s∈Ω with $N_{n}\left(s\right)\geq 1$ and o<κln(ln(n)).

Consider δ>0 and set $\epsilon =\frac{\delta }{{\left|\Delta \right|}^{2o}},$ in Equation (10), then

$$\begin{array}{ccc} \frac{N_{n}\left(s,a\right)}{N_{n}\left(s\right)}-P\left(a|s\right) & \leq & \sqrt{\frac{\delta \ln(\ln(n))}{{\left|\Delta \right|}^{2o}N_{n}\left(s\right)}}\\ N_{n}\left(s,a\right)-N_{n}\left(s\right)P\left(a|s\right) & \leq & \sqrt{\frac{\delta \ln(\ln(n))}{{\left|\Delta \right|}^{2o}}N_{n}\left(s\right)}. \end{array}$$

Because Γ is a good part of P, s∈Γ, we obtain

$$\begin{array}{ccc} \sum_{s\in \Gamma }N_{n}\left(s,a\right)-P\left(a|\Gamma \right)\sum_{s\in \Gamma }N_{n}\left(s\right) & \leq & \sum_{s\in \Gamma }\sqrt{\frac{\delta \ln(\ln(n))}{{\left|\Delta \right|}^{2o}}N_{n}\left(s\right)}. \end{array}$$

Following Equations (3), (4) and (7), we have

$$\begin{array}{ccc} N_{n}\left(\Gamma ,a\right)-P\left(a|\Gamma \right)N_{n}\left(\Gamma \right) & \leq & \frac{\sqrt{\delta \ln(\ln(n))}}{{\left|\Delta \right|}^{o}}\sum_{s\in \Gamma }\sqrt{N_{n}\left(s\right)}, \end{array}$$

then,

$$\begin{array}{ccc} \frac{N_{n}\left(\Gamma ,a\right)}{N_{n}\left(\Gamma \right)}-P\left(a|\Gamma \right) & \leq & \frac{\sqrt{\delta \ln(\ln(n))}}{{\left|\Delta \right|}^{o}N_{n}\left(\Gamma \right)}\left|\Gamma \right|\sqrt{\max_{s\in \Gamma }\left(N_{n}\left(s\right)\right)}\\ & \leq & \frac{\sqrt{\delta \ln(\ln(n))}}{{\left|\Delta \right|}^{o}N_{n}\left(\Gamma \right)}{\left|\Delta \right|}^{o}\sqrt{\sum_{s\in \Gamma }N_{n}\left(s\right)}\\ & = & \frac{\sqrt{\delta \ln(\ln(n))}}{N_{n}\left(\Gamma \right)}\sqrt{N_{n}\left(\Gamma \right)}\\ & = & \sqrt{\frac{\delta \ln(\ln(n))}{N_{n}\left(\Gamma \right)}}. \end{array}$$

□

The next results show that despite ln(ln(n)) violating the second condition imposed by Equation (9), the EDC (with ln(ln(n))) provides a consistent estimate of the minimal partition.

**Theorem** **1.** *Let $\left(X_{t}\right)$ be a Markov chain of order o, with finite and discrete alphabet Δ,o<∞, and state space $\Omega =\Delta^{o}$; $x_{1}^{n}$ is a sample of the process and let $P=\{\Gamma_{1},...,\Gamma_{\left|P\right|}\}$ be a partition of Ω, and suppose that i and j exist; i≠j such that $\Gamma_{i}$ and $\Gamma_{j}$ following Definition 3-i. Then, $P\left(a|\Gamma_{i}\right)=P\left(a|\Gamma_{j}\right),$ ∀a∈Δ if, and only if, eventually, almost surely as n→∞,*

$$EDC\left(x_{1}^{n},P^{ij}\right)>EDC\left(x_{1}^{n},P\right).$$

*where $EDC(x_{1}^{n},P)$ is defined by Equation (8), with $w_{n}=\ln\left(\ln\left(n\right)\right)$ and $EDC(x_{1}^{n},P^{ij})$ is given by Equation (8) (with $w_{n}=\ln\left(\ln\left(n\right)\right)$) over the partition $P^{ij}=\left\{\left\{P\setminus \{\Gamma_{i}\}\right\}\setminus \left\{\Gamma_{j}\right\}\right\}\cup \Gamma_{ij}$ and $\Gamma_{ij}=\left\{\Gamma_{i}\cup \Gamma_{j}\right\}.$*

**Proof.**  The proof is a variant of the one presented in [2], theorem 1. ⇐ is direct from that proof, just considering (i) $\frac{\ln(\ln(n))}{n}\to 0$ instead of $\frac{\ln(n)}{n}\to 0,$ when n→∞ and considering (ii) that γ(·) is an increasing function. For ⇒, we have that $P\left(a|\Gamma_{i}\right)=P\left(a|\Gamma_{j}\right),\forall a\in \Delta ,$ and we want to prove that $EDC\left(x_{1}^{n},P\right)-EDC\left(x_{1}^{n},P^{ij}\right)<0.$ Again, following the steps in such a proof, we obtain that $EDC\left(x_{1}^{n},P\right)-EDC\left(x_{1}^{n},P^{ij}\right)$ is bounded above by

$$\begin{array}{ccc} & & N_{n}\left(\Gamma_{i}\right)D\left(\frac{N_{n}\left(\Gamma_{i},.\right)}{N_{n}\left(\Gamma_{i}\right)}||P\left(.|\Gamma_{i}\right)\right)+N_{n}\left(\Gamma_{j}\right)D\left(\frac{N_{n}\left(\Gamma_{j},.\right)}{N_{n}\left(\Gamma_{j}\right)}||P\left(.|\Gamma_{j}\right)\right)\\ & & -\left(\gamma \left(\frac{\left(|\Delta |-1)|P\right|}{\alpha }\right)-\gamma \left(\frac{\left(|\Delta |-1)(|P|-1\right)}{\alpha }\right)\right)\ln\left(\ln\left(n\right)\right), \end{array}$$

where D(P(·)||Q(·)) is the relative entropy, given by Definition 5.

For each $\Gamma \in \{\Gamma_{i},\Gamma_{j}\},$ $\frac{N_{n}\left(\Gamma ,.\right)}{N_{n}\left(\Gamma \right)}$ and P(.|Γ) are probabilities on Δ; then, Equation (11) follows from lemma 6.3 in [7]. On the other hand, since $\Gamma \in \{\Gamma_{i},\Gamma_{j}\}$ is a good part, by hypothesis, from Proposition 1, for any δ>0 and large enough *n*, Equation (12) follows,

(11) $$\begin{array}{ccc} D\left(\frac{N_{n}\left(\Gamma ,.\right)}{N_{n}\left(\Gamma \right)}||P\left(.|\Gamma \right)\right) & \leq & \sum_{a\in \Delta }\frac{{\left(\frac{N_{n}\left(\Gamma ,a\right)}{N_{n}\left(\Gamma \right)}-P\left(a|\Gamma \right)\right)}^{2}}{P(a|\Gamma )} \end{array}$$

(12) $$\begin{array}{ccc} & \leq & \sum_{a\in \Delta }\frac{\frac{\delta \ln(\ln(n))}{N_{n}\left(\Gamma \right)}}{P(a|\Gamma )}. \end{array}$$

Then, set $c_{0}=\left(\gamma \left(\frac{\left(|\Delta |-1)|P\right|}{\alpha }\right)-\gamma \left(\frac{\left(|\Delta |-1)(|P|-1\right)}{\alpha }\right)\right)$, which is >0, since γ(·) in a strictly increasing function. For any δ>0 and large enough *n*,

$$\begin{array}{ccc} EDC\left(x_{1}^{n},P\right)-EDC\left(x_{1}^{n},P^{ij}\right) & \leq & \frac{2\delta |\Delta |}{p}\ln\left(\ln\left(n\right)\right)-c_{0}\ln\left(\ln\left(n\right)\right)\\ & = & \ln\left(\ln\left(n\right)\right)\left(\frac{2\delta |\Delta |}{p}-c_{0}\right) \end{array}$$

where $p=\min\{P\left(a|\Gamma \right):a\in \Delta ,\Gamma \in \left\{\Gamma_{i},\Gamma_{j}\right\}\}.$ In particular, taking $\delta <\frac{pc_{0}}{2|\Delta |}$, for a large enough *n*, $EDC\left(x_{1}^{n},P\right)-EDC\left(x_{1}^{n},P^{ij}\right)<0.$ □

As a result of the previous theorem, it turns out that it is possible to guarantee that the EDC with the penalty term $w_{n}=\ln\left(\ln\left(n\right)\right)$ allows the consistent estimation of the minimal partition. As a consequence, we have:

**Corollary** **1.** *Let $\left(X_{t}\right)$ be a Markov chain of order o, with finite and discrete alphabet Δ,o<∞, and state space $\Omega =\Delta^{o}$; $x_{1}^{n}$ is a sample of the process. Let* Ψ *be the set of all the partitions of Ω. Define*

$$P_{n}^{*}={argmax}_{P\in \Psi }\left\{EDC\left(x_{1}^{n},P\right)\right\}$$

where $EDC(x_{1}^{n},P)$ is defined by Equation (8), with $w_{n}=\ln\left(\ln\left(n\right)\right).$ Then, eventually, almost surely as n→∞, $P^{*}=P_{n}^{*},$ where $P^{*}$ is the partition of Ω, following Definition 2.

**Proof.**  Following the same steps as the proof of Theorem 3 of [2]. It is enough to replace the BIC criterion with the EDC criterion (Equation (8)) with $w_{n}=\ln\left(\ln\left(n\right)\right)$ and apply Theorem 1 instead of Theorem 1 and Corollary 1 of [2]. □

Corollary 1 complements the results of [4], showing that the minimal partition (Definition 2) is consistently recovered by the EDC (Equation (8)) when it is formulated by a strictly increasing function γ and $w_{n}$ follows Equation (9), or when $w_{n}=\ln\left(\ln\left(n\right)\right).$

In order to generalize the BIC-based metric $d_{P},$ given by Definition 4, the following notion is introduced.

**Definition** **6.** *Let $\left(X_{t}\right)$ be a Markov chain of order o, with finite and discrete alphabet Δ,o<∞, and state space $\Omega =\Delta^{o}$; $x_{1}^{n}$ is a sample of the process, let $P=\{\Gamma_{1},...,\Gamma_{\left|P\right|}\}$ be a good partition of Ω, and 1≤i,j≤|P|,i≠j:*

$$\begin{array}{c} \delta_{P}\left(i,j\right)=v_{n}\sum_{a\in \Delta }\left\{\sum_{k\in \{i,j\}}N_{n}\left(\Gamma_{k},a\right)\ln\left(\frac{N_{n}\left(\Gamma_{k},a\right)}{N_{n}\left(\Gamma_{k}\right)}\right)\right.\left.-N_{n}\left(\Gamma_{ij},a\right)\ln\left(\frac{N_{n}\left(\Gamma_{ij},a\right)}{N_{n}\left(\Gamma_{ij}\right)}\right)\right\}, \end{array}$$

*where $N_{n}\left(\Gamma_{ij}\right)=N_{n}\left(\Gamma_{i}\right)+N_{n}\left(\Gamma_{j}\right),$ $N_{n}\left(\Gamma_{ij},a\right)=N_{n}\left(\Gamma_{i},a\right)+N_{n}\left(\Gamma_{j},a\right),\;\forall a\in \Delta .$ With $v_{n}^{-1}=w_{n}\left(\gamma \left(\frac{\left(|\Delta |-1)|P\right|}{\alpha }\right)-\gamma \left(\frac{\left(|\Delta |-1)(|P|-1\right)}{\alpha }\right)\right),$ α a constant and positive value, γ(·) being a strictly increasing function, and $\left\{w_{n}\right\}$ a sequence of positive numbers depending on n.*

It is evident that if we take $\gamma \left(\frac{\left(|\Delta |-1)|P\right|}{\alpha }\right)=\frac{\left(|\Delta |-1)|P\right|}{\alpha }$ and $w_{n}=\ln\left(n\right),$ Definition 6 coincides with Definition 4.

The next result shows the relationship between the EDC criterion and the notion introduced in Definition 6.

**Theorem** **2.** *Let $\left(X_{t}\right)$ be a Markov chain of order o, with finite and discrete alphabet Δ,o<∞, and $\Omega =\Delta^{o}$; $x_{1}^{n}$ is a sample of the process. Let $P=\{\Gamma_{1},...,\Gamma_{\left|P\right|}\}$ be a good partition of Ω, and 1≤i,j≤|P|,i≠j, then,*

$$\begin{array}{c} EDC\left(x_{1}^{n},P\right)<EDC\left(x_{1}^{n},P^{ij}\right)\Leftrightarrow \delta_{P}\left(i,j\right)<1. \end{array}$$

*where $\delta_{P}\left(i,j\right)$ is given by Definition 6, $EDC(x_{1}^{n},P)$ is defined by Equation (8), and $EDC(x_{1}^{n},P^{ij})$ is given by Equation (8) over the partition $P^{ij}=\left\{\left\{P\setminus \{\Gamma_{i}\}\right\}\setminus \left\{\Gamma_{j}\right\}\right\}\cup \Gamma_{ij}$ and $\Gamma_{ij}=\left\{\Gamma_{i}\cup \Gamma_{j}\right\}.$*

**Proof.**  (13) $$\begin{array}{ccc} \text{EDC}\left(x_{1}^{n},P\right)-\text{EDC}\left(x_{1}^{n},P^{ij}\right) & = & \sum_{a\in \Delta }\sum_{k\in \{i,j\}}N_{n}\left(\Gamma_{k},a\right)\ln\left(\frac{N_{n}\left(\Gamma_{k},a\right)}{N_{n}\left(\Gamma_{k}\right)}\right)-\\ \; & \; & \sum_{a\in \Delta }N_{n}\left(\Gamma_{ij},a\right)\ln\left(\frac{N_{n}\left(\Gamma_{ij},a\right)}{N_{n}\left(\Gamma_{ij`}\right)}\right)-v_{n}^{-1} \end{array}$$

Note that $\text{EDC}\left(x_{1}^{n},P\right)<\text{EDC}\left(x_{1}^{n},P^{ij}\right)\Leftrightarrow \text{EDC}\left(x_{1}^{n},P\right)-\text{EDC}\left(x_{1}^{n},P^{ij}\right)<0,$ and $\text{EDC}(x_{1}^{n},P)$$-\text{EDC}\left(x_{1}^{n},P^{ij}\right)<0\Leftrightarrow \delta_{P}\left(i,j\right)<1,$ applying Equation (13), since $v_{n}>0.$ □

**Remark** **1.** *In order to guarantee the consistent estimation of the partition given by Definition 2, we note that Theorem 2 must be used for a large enough n and with weights $w_{n}$ following Equation (9) or $w_{n}=\ln\left(\ln\left(n\right)\right).$*

The following theorem characterizes the notion given by Definition 6 as being a metric.

**Theorem** **3.** *Let $\left(X_{t}\right)$ be a Markov chain of order o over a finite and discrete alphabet Δ,o<∞, $\Omega =\Delta^{o}$ the state space, and $x_{1}^{n}$ a sample of the Markov process. If $P=\{\Gamma_{1},\ldots ,\Gamma_{\left|P\right|}\}$ is a good partition of* Ω*, for each n, and for any i,j,k∈{1,2,...,|P|}, given $\delta_{P}$ as Definition 6,*

*i*. *$\delta_{P}\left(i,j\right)\geq 0,$ with equality, if and only if, $\frac{N_{n}\left(\Gamma_{i},a\right)}{N_{n}\left(\Gamma_{i}\right)}=\frac{N_{n}\left(\Gamma_{j},a\right)}{N_{n}\left(\Gamma_{j}\right)}\;\;\forall a\in \Delta ;$*
*ii*. $\delta_{P}\left(i,j\right)=\delta_{P}\left(j,i\right);$
*iii*. $\delta_{P}\left(i,k\right)\leq \delta_{P}\left(i,j\right)+\delta_{P}\left(j,k\right).$

**Proof.**  Here, we only prove iii. since item i. is straightforward from Theorem 2 [2] and ii. follows from definition. Consider the relative entropy between two probabilities *P* and *Q* on the alphabet Δ, $D\left(P\left(\cdot \right)||Q\left(\cdot \right)\right)=\sum_{a\in \Delta }P\left(a\right)\ln\left(P\left(a\right)/Q\left(a\right)\right).$ *D* is non-negative, furthermore *D* is zero if and only if P(·)=Q(·). Returning to our goal, iii. occurs if and only if

(14) $$0\leq v_{n}^{-1}\left(\delta_{P}\left(i,j\right)+\delta_{P}\left(j,k\right)-\delta_{P}\left(i,k\right)\right)=\left(*\right)$$

since $v_{n}>0.$ We inspect the right side of Equation (14),

$$\begin{array}{ccc} \left(*\right) & = & \sum_{s=i,k}\sum_{a\in \Delta }N_{n}\left(\Gamma_{j},a\right)\left(\ln\left(\frac{N_{n}\left(\Gamma_{j},a\right)}{N_{n}\left(\Gamma_{j}\right)}\right)-\ln\left(\frac{N_{n}\left(\Gamma_{js},a\right)}{N_{n}\left(\Gamma_{js}\right)}\right)\right)+\\ & & \sum_{s=i,k}\sum_{a\in \Delta }N_{n}\left(\Gamma_{s},a\right)\left(\ln\left(\frac{N_{n}\left(\Gamma_{ik},a\right)}{N_{n}\left(\Gamma_{ik}\right)}\right)-\ln\left(\frac{N_{n}\left(\Gamma_{sj},a\right)}{N_{n}\left(\Gamma_{sj}\right)}\right)\right)\\ & = & \sum_{s=i,k}N_{n}\left(\Gamma_{j}\right)\sum_{a\in \Delta }\frac{N_{n}\left(\Gamma_{j},a\right)}{N_{n}\left(\Gamma_{j}\right)}\ln\left(\frac{N_{n}\left(\Gamma_{j},a\right)}{N_{n}\left(\Gamma_{j}\right)}/\frac{N_{n}\left(\Gamma_{js},a\right)}{N_{n}\left(\Gamma_{js}\right)}\right)+\\ & & \sum_{s=i,k}\sum_{a\in \Delta }N_{n}\left(\Gamma_{s},a\right)\ln\left(\frac{N_{n}\left(\Gamma_{ik},a\right)}{N_{n}\left(\Gamma_{ik}\right)}/\frac{N_{n}\left(\Gamma_{sj},a\right)}{N_{n}\left(\Gamma_{sj}\right)}\right)\\ & {=}^{\left(1\right)} & N_{n}\left(\Gamma_{j}\right)\sum_{s=i,k}D\left(\frac{N_{n}\left(\Gamma_{j},\cdot \right)}{N_{n}\left(\Gamma_{j}\right)}\left|\right|\frac{N_{n}\left(\Gamma_{js},\cdot \right)}{N_{n}\left(\Gamma_{js}\right)}\right)+\\ & & \sum_{s=i,k}\sum_{a\in \Delta }N_{n}\left(\Gamma_{s},a\right)\frac{N_{n}\left(\Gamma_{ik}\right)}{N_{n}\left(\Gamma_{ik},a\right)}\frac{N_{n}\left(\Gamma_{ik},a\right)}{N_{n}\left(\Gamma_{ik}\right)}\ln\left(\frac{N_{n}\left(\Gamma_{ik},a\right)}{N_{n}\left(\Gamma_{ik}\right)}/\frac{N_{n}\left(\Gamma_{sj},a\right)}{N_{n}\left(\Gamma_{sj}\right)}\right)\\ & \geq^{\left(2\right)} & N_{n}\left(\Gamma_{j}\right)\sum_{s=i,k}D\left(\frac{N_{n}\left(\Gamma_{j},\cdot \right)}{N_{n}\left(\Gamma_{j}\right)}\left|\right|\frac{N_{n}\left(\Gamma_{js},\cdot \right)}{N_{n}\left(\Gamma_{js}\right)}\right)+\sum_{s=i,k}\frac{1}{n}D\left(\frac{N_{n}\left(\Gamma_{ik},\cdot \right)}{N_{n}\left(\Gamma_{ik}\right)}\left|\right|\frac{N_{n}\left(\Gamma_{sj},\cdot \right)}{N_{n}\left(\Gamma_{sj}\right)}\right)\geq^{\left(3\right)}0. \end{array}$$

where (1) follows from the definition of the relative entropy D, between two empirical laws $\frac{N_{n}\left(\Gamma_{j},\cdot \right)}{N_{n}\left(\Gamma_{j}\right)}$ and $\frac{N_{n}\left(\Gamma_{js},\cdot \right)}{N_{n}\left(\Gamma_{js}\right)}.$ (2) follows from $\frac{1}{N_{n}\left(\Gamma_{ik},a\right)}\geq \frac{1}{n}$ and $N_{n}\left(\Gamma_{s},a\right)N_{n}\left(\Gamma_{ik}\right)\geq 1,$ and using the relative entropy *D* between the two empirical laws $\frac{N_{n}\left(\Gamma_{ik},\cdot \right)}{N_{n}\left(\Gamma_{ik}\right)}$ and $\frac{N_{n}\left(\Gamma_{sj},\cdot \right)}{N_{n}\left(\Gamma_{sj}\right)}.$ The last inequality (3) is valid since *D* is non-negative. Then, Equation (14) is valid. □

We saw by Theorem 2 that the improvement in the construction of the partition, when joining the parts $\Gamma_{i}$ and $\Gamma_{j}$ is detected by the metric $\delta_{P}$ when it takes a value less than 1, a value that could be used as a reference. It is clear that this property should only be used for large enough values of *n*, and a penalization term following Equation (9), according to [4], or when $w_{n}=\ln\left(\ln\left(n\right)\right)$ according to Theorem 1 and Corollary 1, which is when the EDC criterion is capable of consistently estimating the partition that follows Definition 2.

The following section shows an example of applying the metric—Definition 6—to real data. We seek to show how the model—Definition 2—varies by varying the penalty term $w_{n}$ for cases where the consistency of the estimate is guaranteed, when *n* is large enough, that is, with terms $w_{n}$ following Remark 1.

In the application we consider genetic sequences of dengue virus in FASTA format. In [8], a variant of the model specified in Definition 2 is designed specifically to model the first DNA sequence (FASTA format) of SARS-CoV-2 virus, Genbank number MN908947 (accessible at: https://www.ncbi.nlm.nih.gov/nuccore/MN908947, accessed on 10 March 2024). Furthermore, partition Markov models have been instrumental in modeling the DNA of the SARS-CoV-2 virus, exposing the evolution of the various variants during the pandemic period (see, for example, [9]).

## 3. Application

We examine and model three DNA sequences sourced from dengue virus type 3 (DENV-3), originating from Brazil. These sequences were sequenced and made publicly available in early 2023 (https://www.ncbi.nlm.nih.gov/, accessed on 10 March 2024). We then proceed to compare the models derived from these sequences by applying the metric—Definition 6—and employing the agglomerative algorithm. Our analysis focuses on observing the variations in partition composition and probability magnitudes as we change the penalization term $w_{n}.$

According to [10], the genesis of the initial autochthonous case of DENV-3 (GIII-American-I lineage) in Brazil dates back to December 2000, specifically within Rio de Janeiro. Over the course of the 2000s multiple incursions of this lineage were documented from the Caribbean into Brazil. The northern and southeastern regions of Brazil swiftly emerged as the epicenters of dissemination. The advent of this lineage precipitated a significant dengue outbreak in Brazil, in Rio de Janeiro, in 2002, followed by subsequent outbreaks in diverse locales.

However, since 2010, publicly available data indicate a downward tendency in the prevalence of DENV-3; DENV-3 has represented a mere fraction (<1%) of the total dengue cases in Brazil, with scant confirmed instances reported. Consequently, the transmission of DENV-3 has not been substantiated in recent years, pointing out a potential extinction of the DENV-3 (GIII-American-I lineage) within Brazil. The resurgence of DENV-3 is a real challenge in Brazil, since it is expected that the population will not have immunity, given the time that this virus has not been found in the region.

Table 1 shows the GenBank numbers, collection date, and origins of three sequences, introduced by [10]. The records correspond to three complete genetic sequences in FASTA format (alphabet Δ={a,c,g,t}), of DENV-3, which is already native to Brazil.

We assume that each of these sequences is a sample of a process that meets Definition 2. We proceed to fit the model—Definition 2—using the metric—Definition 6—and the agglomerative algorithm. For this, we take into account the alphabet Δ={a,c,g,t}, with cardinal |Δ|=4, where the sequences take their values. In Table 2, we show the frequencies for each element of the alphabet.

Considering that min{10,697, 10,511, 10,553} = 10,511 and $\log_{\left|\Delta \right|}$(10,511) = 6.68, with integer part equal to 6, we adopt o=3, since 3<6 and the elements of the genetic alphabet Δ are organized in multiples of 3.

We fit four scenarios for each of the three sequences OQ706226, OQ706227, and OQ706228, with each scenario governed by a different penalty $w_{n}.$ All of them are considered in Definition 6, Δ={a,c,g,t},o=3,α=2 (see [11]), and the γ function is the identity function. For each penalty, we identify, using the metric (Definition 6), the estimated partition of the partition given by Definition 2, and then, determine the transition probabilities of each part for each element of Δ. We denote by $\Gamma_{i}^{v}$ the part *i* estimated for the sequence *v*, where *v* can be A,B,C, corresponding to OQ706226, OQ706227, and OQ706228, respectively. In Table 3 and Table 4, we record the results for the three sequences with penalty $w_{n}=n^{1/2}$; Table 5 and Table 6 report the results for the three sequences with penalty $w_{n}=n^{1/3}.$ While Table 7 and Table 8 show, for the three sequences, the results using the usual BIC penalty ($w_{n}=\ln\left(n\right)$). Finally, Table 9, Table 10 and Table 11 report the results with the penalty $w_{n}=\ln\left(\ln\left(n\right)\right)$ (see Corollary 1): for the sequence OQ706226, Table 9; for the sequence OQ706227, Table 10; and for the sequence OQ706228, Table 11.

We observe from Table 3, Table 5, Table 7 and Table 9 (right)–Table 11 (right) that as the penalty $w_{n}$ is reduced (that is, when $w_{n}$ approaches the lower limit ln(ln(n))), the model is allowed to acquire more parameters, in this case, more parts.

Given a penalization $w_{n},$ the three sequences, *A* (GenBank OQ706226), *B* (GenBank OQ706227), and *C* (GenBank OQ706228), show a similar number of parts. More specifically, for the penalty $w_{n}=n^{1/2}$ the behavior of the three sequences is represented by two parts (Table 3), for $w_{n}=n^{1/3}$ the behavior of the three sequences is described by four parts (Table 5). For $w_{n}=\ln\left(n\right),$ OQ706226 is modeled by five parts while the other two are modeled by six parts (see Table 7). For penalty $w_{n}=\ln\left(\ln\left(n\right)\right),$ OQ706226 is modeled by a partition with 13 parts (see Table 9, right) while the other two sequences are modeled by 14 parts, see Table 10 (right) and Table 11 (right).

The formal determination of whether the identified models, under each penalty, exhibit significant differences lies beyond the scope of this application. However, we acknowledge it as an open question worthy of further exploration.

The following observation applies to all three sequences. Observing the magnitudes of the transition probabilities, marked in bold, we note that, as reported in Table 6, Table 8, and Table 9 (left)–Table 11 (left), there is a predominant number of parts whose prevalence is the transition to element a of the alphabet {a,c,g,t}, and secondly, parts that indicate a prevalence for element g of the alphabet. As for Table 4, which reports the most penalized case ($w_{n}=n^{1/2}$), one part is recorded with prevalence for a and another with prevalence for g, which is natural, since the model only has two parts. As Table 9, Table 10 and Table 11 show, based on the penalty $w_{n}=\ln\left(\ln\left(n\right)\right),$ the three sequences show the same part {agc,ggt,gag,ata} with a prevalence for the element t of the alphabet Δ, of lower magnitude, than those previously mentioned.

## 4. Conclusions

The main objective of this paper, developed in Section 2, is to introduce a new notion based on the Equation (8), as given in Definition 6. This concept is used to identify the minimal partition of a Markov chain—Definition 2. Theorem 3 proves that the concept in Definition 6 constitutes a metric. Furthermore, Theorem 2 establishes the relationship between this new metric and the operation of the EDC criterion, showing that in an iterative process, selecting a partition with a higher EDC value is equivalent to using the value 1 as a threshold in the metric. In this way, we achieve our main goal of proposing an EDC-based metric to estimate the minimal partition.

Our results add to those of [4], in the search to characterize penalty terms that can be used in the EDC criterion to obtain the consistent estimation of the minimal partition. Ref. [4] demonstrates that the EDC, under certain conditions on the term $w_{n}$ (Equation (9)), provides a strongly consistent estimate of the minimal partition, as defined in Definition 2. Building on the results from [5], we conjectured that using $w_{n}=\ln\left(\ln\left(n\right)\right)$ might preserve strong consistency, even though this term does not satisfy the second condition imposed by Equation (9). We confirm in Theorem 1 and Corollary 1 that strong consistency is indeed achieved using the EDC with the penalization term $w_{n}=\ln\left(\ln\left(n\right)\right).$

We conclude the article with an application demonstrating the effect of the metric introduced in Definition 6 on estimating the minimal partition—Definition 2, using various penalty terms discussed in Remark 1. For this purpose, we analyze three Dengue virus type 3 sequences, native to Brazil and collected in 2023, in FASTA format. The application shows that relaxing the penalty results in higher cardinalities for the estimated partition. We identify which parts (collections of states) of the Dengue sequences have a greater or lesser preference for transitioning to the next element (a, c, g, or t) in the alphabet Δ={a,c,g,t}. As expected, the models identified for each sequence exhibit similar features when the penalty is applied, which is natural given that the sequences share the same collection date and region of origin.

## Figures and Tables

**Table 1:** Three autochthonous sequences of DENV-3 in FASTA format, Δ={a,c,g,t}.

Origin	Collection Date	GenBank	Sequence Nickname	Size
Roraima, Canta (Brazil)	4 March 2023	OQ706226	*A*	10,697
Roraima, Boa Vista (Brazil)	22 January 2023	OQ706227	*B*	10,511
Roraima, Boa Vista (Brazil)	3 January 2023	OQ706228	*C*	10,553

**Table 2:** Frequencies $N_{n}\left(a\right),a\in \Delta =\left\{a,c,g,t\right\}.$

GenBank	Sequence Nickname	$N_{n}\left(a\right)$	$N_{n}\left(c\right)$	$N_{n}\left(g\right)$	$N_{n}\left(t\right)$
OQ706226	*A*	3435	2209	2782	2271
OQ706227	*B*	3378	2164	2736	2233
OQ706228	*C*	3395	2173	2743	2242

**Table 3:** Minimal partition—Definition 2—estimated by Definition 6. From top to bottom, for the sequences *A* (GenBank OQ706226), *B* (GenBank OQ706227), and *C* (GenBank OQ706228). $\Delta =\left\{a,c,g,t\right\},o=3,w_{n}=n^{1/2},\alpha =2,$ γ function given by the identity.

Sequence	Part	States
OQ706226	$\Gamma_{1}^{A}$	ccc, tgc, cgc, ggc, gac, gtc, acg, att, gct, gcc, atc, agc, ggt, gag, ata, tcc, agt, tct, ctc, ttc,
		cct, ctt, gtt, aaa, ttt, aag, ccg, gcg, tgg, gtg, tat, agg, ctg, gat, ttg, aat, tcg, cgg, cat, ggg,
		act, atg
	$\Gamma_{2}^{A}$	cag, cac, taa, cgt, aga, gga, tta, tac, cga, acc, tag, gta, cca, tga, tca, cta, gca, aac, tgt, gaa,
		caa, aca
OQ706227	$\Gamma_{1}^{B}$	ccc, ggc, cgc, gac, gtc, acg, gct, tag, gcc, atc, agc, ggt, gag, ata, att, tcc, agt, tgc, tct, gtt,
		ctc, ttc, cct, ctt, aaa, ttt, aag, ccg, gcg, tgg, gtg, tat, agg, ctg, ttg, aat, gat, tcg, cgg, cat,
		act, ggg, atg
	$\Gamma_{2}^{B}$	cag, cgt, aga, gga, tta, tac, cga, cac, acc, taa, tga, cta, cca, tca, gca, gta, aac, tgt, gaa, caa,
		aca
OQ706228	$\Gamma_{1}^{C}$	ccc, cgc, ggc, gac, tgc, gct, tcc, tct, agt, gtc, acg, att, tag, gcc, atc, agc, ggt, gag, ata, ctc,
		cct, ttc, aaa, ctt, gtt, ttt, aag, ccg, gcg, tgg, gtg, agg, ctg, ttg, aat, gat, tcg, cat, tat, cgg,
		ggg, atg, act
	$\Gamma_{2}^{C}$	cag, cgt, tta, cac, aga, gga, cga, tac, acc, taa, tga, cta, gca, cca, tca, gta, aac, tgt, gaa, caa,
		aca

**Table 4:** Transition probabilities—Equation (2)—estimated by Equation (5). From top to bottom, for the sequences *A* (GenBank OQ706226), *B* (GenBank OQ706227), and *C* (GenBank OQ706228). $\Delta =\left\{a,c,g,t\right\},w_{n}=n^{1/2}$; full estimated partitions displayed in Table 3. In bold, the highest probability per part.

OQ706226	*i*	$P(a|\Gamma_{i}^{A})$	$P(c|\Gamma_{i}^{A})$	$P(g|\Gamma_{i}^{A})$	$P(t|\Gamma_{i}^{A})$
	1	**0.37032**	0.21336	0.21256	0.20376
	2	0.20595	0.19061	**0.37152**	0.23192
OQ706227	*i*	$P(a|\Gamma_{i}^{B})$	$P(c|\Gamma_{i}^{B})$	$P(g|\Gamma_{i}^{B})$	$P(t|\Gamma_{i}^{B})$
	1	**0.36846**	0.21441	0.21374	0.20339
	2	0.20723	0.18508	**0.37341**	0.23428
OQ706228	*i*	$P(a|\Gamma_{i}^{C})$	$P(c|\Gamma_{i}^{C})$	$P(g|\Gamma_{i}^{C})$	$P(t|\Gamma_{i}^{C})$
	1	**0.36871**	0.21373	0.21387	0.20369
	2	0.20760	0.18681	**0.37167**	0.23392

**Table 5:** Minimal partition—Definition 2—estimated by Definition 6. From top to bottom, for the sequences *A* (GenBank OQ706226), *B* (GenBank OQ706227), and *C* (GenBank OQ706228). $\Delta =\left\{a,c,g,t\right\},o=3,w_{n}=n^{1/3},\alpha =2,$ γ function given by the identity.

Sequence	Part	States
OQ706226	$\Gamma_{1}^{A}$	ccc, tgc, cgc, ggc, gac, gtc, acg, att, gct, gcc, atc, agc, ggt, gag, ata
	$\Gamma_{2}^{A}$	tcc, tct, agt, ctc, ttc, cct, ctt, gtt, aaa, ttt
	$\Gamma_{3}^{A}$	aag, ccg, gcg, tgg, gtg, agg, ctg, gat, ttg, aat, tcg, cgg, cat, ggg, tat, act, atg
	$\Gamma_{4}^{A}$	cag, cac, taa, cta, cgt, aga, gga, tta, tac, cga, acc, tag, gta, cca, tga, tca, aac, tgt, gaa, caa,
		aca, gca
OQ706227	$\Gamma_{1}^{B}$	ccc, tgc, tct, gtt, cgc, ggc, gac, gtc, gct, acg, att, tag, gcc, atc, agc, ggt, gag, ata
	$\Gamma_{2}^{B}$	tcc, agt, ctc, ttc, cct, ctt, aaa, ttt
	$\Gamma_{3}^{B}$	aag, ccg, gcg, tgg, gtg, agg, ctg, ttg, aat, gat, tcg, cgg, cat, tat, act, ggg, atg
	$\Gamma_{4}^{B}$	cag, cgt, aga, gga, tta, tac, cga, cac, acc, tgt, gaa, caa, taa, tga, cta, cca, tca, aca, gca, gta,
		aac
OQ706228	$\Gamma_{1}^{C}$	ccc, cgc, ggc, gac, tcc, tct, agt, tgc, gct, gtc, acg, att, gcc, atc, agc, ggt, gag, ata
	$\Gamma_{2}^{C}$	ctc, cct, ttc, aaa, ctt, gtt, ttt
	$\Gamma_{3}^{C}$	aag, ccg, gcg, tat, tgg, gtg, agg, ctg, ttg, aat, gat, tcg, cat, cgg, act, ggg, atg
	$\Gamma_{4}^{C}$	cag, taa, tta, cac, cgt, cga, tac, acc, aga, gga, tag, gta, cca, tca, tga, cta, gca, aac, tgt, gaa,
		caa, aca

**Table 6:** Transition probabilities—Equation (2)—estimated by Equation (5). From top to bottom, for the sequences *A* (GenBank OQ706226), *B* (GenBank OQ706227), and *C* (GenBank OQ706228). $\Delta =\left\{a,c,g,t\right\},w_{n}=n^{1/3}$; full estimated partitions displayed in Table 5. In bold, the highest probability per part.

OQ706226	*i*	$P(a|\Gamma_{i}^{A})$	$P(c|\Gamma_{i}^{A})$	$P(g|\Gamma_{i}^{A})$	$P(t|\Gamma_{i}^{A})$
	1	**0.29882**	0.22627	0.25608	0.21882
	2	**0.40166**	0.18760	0.25987	0.15087
	3	**0.41291**	0.22673	0.11709	0.24328
	4	0.20595	0.19061	**0.37152**	0.23192
OQ706227	*i*	$P(a|\Gamma_{i}^{B})$	$P(c|\Gamma_{i}^{B})$	$P(g|\Gamma_{i}^{B})$	$P(t|\Gamma_{i}^{B})$
	1	**0.30233**	0.22596	0.25963	0.21208
	2	**0.40612**	0.18593	0.25902	0.14893
	3	**0.41410**	0.22668	0.11608	0.24314
	4	0.20723	0.18508	**0.37341**	0.23428
OQ706228	*i*	$P(a|\Gamma_{i}^{C})$	$P(c|\Gamma_{i}^{C})$	$P(g|\Gamma_{i}^{C})$	$P(t|\Gamma_{i}^{C})$
	1	**0.31038**	0.22205	0.26436	0.20321
	2	**0.42182**	0.17773	0.24578	0.15467
	3	**0.41362**	0.22573	0.11644	0.24422
	4	0.20761	0.19017	**0.37147**	0.23075

**Table 7:** Minimal partition—Definition 2—estimated by Definition 6. From top to bottom, for the sequences *A* (GenBank OQ706226), *B* (GenBank OQ706227), and *C* (GenBank OQ706228). $\Delta =\left\{a,c,g,t\right\},o=3,w_{n}=\ln\left(n\right),\alpha =2,$ γ function given by the identity.

Sequence	Part	States
OQ706226	$\Gamma_{1}^{A}$	ccc, tgc, cgc, ggc, gac, gtc, acg, att, gct, gcc, atc, agc, ggt, gag, ata
	$\Gamma_{2}^{A}$	tcc, agt, ctc, tct, ttc, cct, ctt, gtt, aaa, ttt
	$\Gamma_{3}^{A}$	aag, ccg, gcg, tgg, gtg, act, agg, ctg, gat, ttg, aat, tcg, cgg, cat, ggg, tat, atg
	$\Gamma_{4}^{A}$	cag, cac, taa, cgt, aga, gga, tta, tac, cga, acc, tag, gta, cca, tca, tga, cta, aac
	$\Gamma_{5}^{A}$	tgt, gaa, caa, aca, gca
OQ706227	$\Gamma_{1}^{B}$	ccc, tgc, tct, gtt, cgc, ggc, gac, gtc, gct, acg, att, tag
	$\Gamma_{2}^{B}$	gcc, atc, agc, ggt, gag, ata
	$\Gamma_{3}^{B}$	tcc, agt, ctc, ttc, cct, ctt, aaa, ttt
	$\Gamma_{4}^{B}$	aag, ccg, gcg, tgg, gtg, agg, ctg, ttg, aat, gat, tcg, cgg, cat, tat, act, ggg, atg
	$\Gamma_{5}^{B}$	cag, cgt, aga, gga, tta, tac, cga, cac, acc
	$\Gamma_{6}^{B}$	tgt, gaa, caa, taa, tga, cta, cca, tca, aca, gca, gta, aac
OQ706228	$\Gamma_{1}^{C}$	ccc, cgc, ggc, gac, tcc, tct, agt, tgc, gct, gtc, acg, att
	$\Gamma_{2}^{C}$	gcc, atc, agc, ggt, gag, ata
	$\Gamma_{3}^{C}$	ctc, cct, ttc, aaa, ctt, gtt, ttt
	$\Gamma_{4}^{C}$	aag, ccg, gcg, tgg, gtg, agg, ctg, ttg, aat, gat, tcg, cgg, cat, tat, act, ggg, atg
	$\Gamma_{5}^{C}$	cag, taa, tta, cac, cgt, cga, tac, acc, aga, gga
	$\Gamma_{6}^{C}$	tag, gta, cca, tca, tga, cta, gca, aac, tgt, gaa, caa, aca

**Table 8:** Transition probabilities—Equation (2)—estimated by Equation (5). From top to bottom, for the sequences *A* (GenBank OQ706226), *B* (GenBank OQ706227), and *C* (GenBank OQ706228). $\Delta =\left\{a,c,g,t\right\},w_{n}=\ln\left(n\right)$; full estimated partitions displayed in Table 7. In bold, the highest probability per part.

OQ706226	*i*	$P(a|\Gamma_{i}^{A})$	$P(c|\Gamma_{i}^{A})$	$P(g|\Gamma_{i}^{A})$	$P(t|\Gamma_{i}^{A})$
	1	**0.29882**	0.22627	0.25608	0.21882
	2	**0.40166**	0.18760	0.25987	0.15087
	3	**0.41291**	0.22673	0.11709	0.24328
	4	0.18351	0.20596	**0.35967**	0.25086
	5	0.26507	0.15017	**0.40273**	0.18203
OQ706227	*i*	$P(a|\Gamma_{i}^{B})$	$P(c|\Gamma_{i}^{B})$	$P(g|\Gamma_{i}^{B})$	$P(t|\Gamma_{i}^{B})$
	1	**0.30945**	0.21394	0.28167	0.19493
	2	**0.28468**	0.25573	0.20507	0.25452
	3	**0.40612**	0.18593	0.25902	0.14893
	4	**0.41410**	0.22668	0.11608	0.24314
	5	0.18868	0.20926	**0.31990**	0.28216
	6	0.21860	0.17026	**0.40620**	0.20494
OQ706228	*i*	$P(a|\Gamma_{i}^{C})$	$P(c|\Gamma_{i}^{C})$	$P(g|\Gamma_{i}^{C})$	$P(t|\Gamma_{i}^{C})$
	1	**0.31933**	0.21040	0.28482	0.18545
	2	**0.28451**	0.25570	0.20528	0.25450
	3	**0.42182**	0.17773	0.24578	0.15467
	4	**0.41362**	0.22573	0.11644	0.24422
	5	0.19202	0.20975	**0.31905**	0.27917
	6	0.21932	0.17546	**0.41088**	0.19434

**Table 9:** Sequence OQ706226. Right: Minimal partition—Definition 2—estimated by Definition 6. Left: Transition probabilities—Equation (2)—estimated by Equation (5); in bold, the highest probability per part. Δ={a,c,g,t},o=3,γ function given by the identity, $w_{n}=\ln\left(\ln\left(n\right)\right).$

*i*	$P(a|\Gamma_{i}^{A})$	$P(c|\Gamma_{i}^{A})$	$P(g|\Gamma_{i}^{A})$	$P(t|\Gamma_{i}^{A})$	Part	States
1	**0.31140**	0.19342	0.28411	0.21107	$\Gamma_{1}^{A}$	ccc, tgc, cgc, ggc, gac
2	**0.32218**	0.25314	0.19456	0.23013	$\Gamma_{2}^{A}$	gcc, atc
3	**0.38732**	0.19249	0.27934	0.14085	$\Gamma_{3}^{A}$	tcc, tct, agt, ctc, ttc, cct
4	0.23757	0.25967	0.21823	**0.28453**	$\Gamma_{4}^{A}$	agc, ggt, gag, ata
5	**0.28879**	0.26078	0.27371	0.17672	$\Gamma_{5}^{A}$	gtc, acg, att, gct
6	**0.40686**	0.21316	0.14272	0.23726	$\Gamma_{6}^{A}$	aag, ccg, gcg, tgg, gtg, ttg, tat
7	0.19113	0.20930	**0.32195**	0.27762	$\Gamma_{7}^{A}$	cag, cac, taa, cgt, aga, gga, tta, tac, cga, acc
8	0.17164	0.29104	**0.41791**	0.11940	$\Gamma_{8}^{A}$	tag, gta
9	**0.46675**	0.21455	0.08532	0.23337	$\Gamma_{9}^{A}$	tcg, cgg, cat, ggg, act, atg
10	**0.34566**	0.27172	0.11275	0.26987	$\Gamma_{10}^{A}$	agg, ctg, gat, aat
11	0.26507	0.15017	**0.40273**	0.18203	$\Gamma_{11}^{A}$	tgt, gaa, caa, aca, gca
12	**0.43116**	0.17754	0.21981	0.17150	$\Gamma_{12}^{A}$	ctt, gtt, aaa, ttt
13	0.17246	0.18610	**0.41439**	0.22705	$\Gamma_{13}^{A}$	cca, tca, tga, cta, aac

**Table 10:** Sequence OQ706227. Right: Minimal partition—Definition 2—estimated by Definition 6. Left: Transition probabilities—Equation (2)—estimated by Equation (5); in bold, the highest probability per part. Δ={a,c,g,t},o=3,γ function given by the identity, $w_{n}=\ln\left(\ln\left(n\right)\right).$

*i*	$P(a|\Gamma_{i}^{B})$	$P(c|\Gamma_{i}^{B})$	$P(g|\Gamma_{i}^{B})$	$P(t|\Gamma_{i}^{B})$	Part	States
1	**0.34479**	0.19313	0.27725	0.18483	$\Gamma_{1}^{B}$	ccc, tgc, gtt, tct
2	**0.31992**	0.25636	0.19280	0.23093	$\Gamma_{2}^{B}$	gcc, atc
3	**0.39127**	0.19183	0.27978	0.13712	$\Gamma_{3}^{B}$	tcc, agt, ctc, ttc, cct
4	0.23810	0.25490	0.22129	**0.28571**	$\Gamma_{4}^{B}$	agc, ggt, gag, ata
5	**0.29094**	0.19737	0.28509	0.22661	$\Gamma_{5}^{B}$	cgc, ggc, gac
6	0.27672	0.26908	**0.28435**	0.16985	$\Gamma_{6}^{B}$	gtc, gct, acg, att, tag
7	**0.40705**	0.21628	0.14945	0.22722	$\Gamma_{7}^{B}$	aag, ccg, gcg, tgg, gtg
8	0.18868	0.20926	**0.31990**	0.28216	$\Gamma_{8}^{B}$	cag, cgt, aga, gga, tta, tac, cga, cac, acc
9	**0.47018**	0.20642	0.08601	0.23739	$\Gamma_{9}^{B}$	tcg, cgg, cat, tat, act, ggg, atg
10	**0.35015**	0.26558	0.11424	0.27003	$\Gamma_{10}^{B}$	agg, ctg, ttg, aat, gat
11	0.27992	0.13996	**0.42191**	0.15822	$\Gamma_{11}^{B}$	tgt, gaa, caa
12	**0.43490**	0.17450	0.21880	0.17181	$\Gamma_{12}^{B}$	ctt, aaa, ttt
13	0.21127	0.17647	**0.39188**	0.22038	$\Gamma_{13}^{B}$	taa, tga, cta, cca, tca, aca, gca
14	0.11330	0.20690	**0.45321**	0.22660	$\Gamma_{14}^{B}$	gta, aac

**Table 11:** Sequence OQ706228. Right: Minimal partition—Definition 2—estimated by Definition 6. Left: Transition probabilities—Equation (2)—estimated by Equation (5); in bold, the highest probability per part. Δ={a,c,g,t},o=3,γ function given by the identity, $w_{n}=\ln\left(\ln\left(n\right)\right).$

*i*	$P(a|\Gamma_{i}^{C})$	$P(c|\Gamma_{i}^{C})$	$P(g|\Gamma_{i}^{C})$	$P(t|\Gamma_{i}^{C})$	Part	States
1	**0.30740**	0.18975	0.28463	0.21822	$\Gamma_{1}^{C}$	ccc, cgc, ggc, gac
2	**0.31924**	0.25581	0.19450	0.23044	$\Gamma_{2}^{C}$	gcc, atc
3	**0.35319**	0.20914	0.29501	0.14266	$\Gamma_{3}^{C}$	tcc, tct, agt
4	0.23889	0.25556	0.21944	**0.28611**	$\Gamma_{4}^{C}$	agc, ggt, gag, ata
5	**0.30048**	0.24642	0.27345	0.17965	$\Gamma_{5}^{C}$	tgc, gct, gtc, acg, att
6	**0.41354**	0.17708	0.26875	0.14063	$\Gamma_{6}^{C}$	ctc, ttc, cct
7	**0.41304**	0.21196	0.14565	0.22935	$\Gamma_{7}^{C}$	aag, ccg, gcg, tat, tgg, gtg
8	0.19202	0.20975	**0.31905**	0.27917	$\Gamma_{8}^{C}$	cag, taa, tta, cac, cgt, cga, tac, acc, aga, gga
9	0.17778	0.28889	**0.41481**	0.11852	$\Gamma_{9}^{C}$	tag, gta
10	**0.47315**	0.20716	0.08440	0.23529	$\Gamma_{10}^{C}$	tcg, cat, cgg, act, ggg, atg
11	**0.34564**	0.26588	0.11374	0.27474	$\Gamma_{11}^{C}$	agg, ctg, ttg, aat, gat
12	0.27205	0.15097	**0.41555**	0.16144	$\Gamma_{12}^{C}$	tgt, gaa, caa, aca
13	**0.43154**	0.17848	0.21883	0.17115	$\Gamma_{13}^{C}$	ctt, gtt, aaa, ttt
14	0.18957	0.17653	**0.40722**	0.22668	$\Gamma_{14}^{C}$	cca, tca, tga, cta, gca, aac

## Data Availability

The National Center for Biotechnology Information Advances Science and Health, https://www.ncbi.nlm.nih.gov/ (accessed on 10 March 2024).
